# High-detectivity perovskite-based photodetector using a Zr-doped TiO_*x*_ cathode interlayer†

**DOI:** 10.1039/c8ra00730f

**Published:** 2018-02-22

**Authors:** C. H. Ji, K. T. Kim, S. Y. Oh

**Affiliations:** Department of Chemical and Biomolecular Engineering, Sogang University Seoul Korea syoh@sogang.ac.kr +82-2-714-3890 +82-2-705-8681

## Abstract

We investigated the incorporation of Zr into TiO_*x*_ cathode interlayers used as hole-blocking layers in an organometallic halide perovskite-based photodetector. The device configuration is ITO/PEDOT:PSS/CH_3_NH_3_PbI_*x*_Cl_3−*x*_/PC_60_BM/Zr–TiO_*x*_/Al. The use of Zr–TiO_*x*_ in the perovskite photodetector reduces the leakage current and improves carrier extraction. The performance of the perovskite photodetector was confirmed by analyzing the current–voltage characteristics, impedance behaviors, and dynamic characteristics. The device with a Zr–TiO_*x*_ layer has a high specific detectivity of 1.37 × 10^13^ Jones and a bandwidth of 2.1 MHz at a relatively low reverse bias and light intensity. Therefore, it can be effectively applied to devices such as image and optical sensors.

## Abstract 2

The use of Zr–TiO_*x*_ in the perovskite photodetector reduces the leakage current and improves carrier extraction.

## Graphical abstract

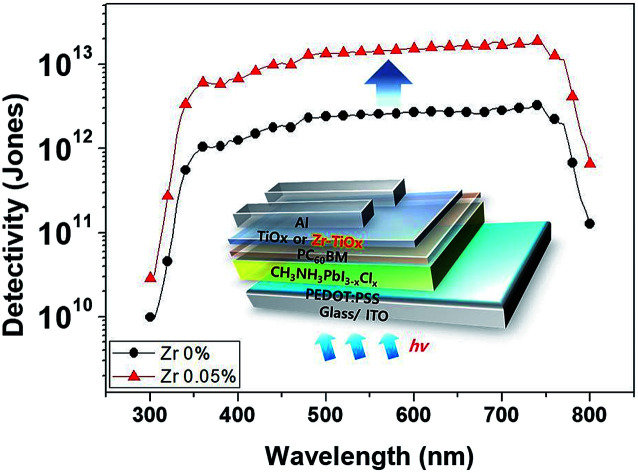

## Introduction

Organometallic halide perovskites of the form CH_3_NH_3_PbX_3−*x*_Y_*x*_ (MAPbX_3−*x*_Y_*x*_), where X and Y denote halides such as I, Br, and Cl, have excellent semiconducting properties, such as broad absorption ranges, small exciton binding energies (∼20 meV), and long charge diffusion lengths (100–1000 nm).^1–3^ Therefore, they have achieved certified photovoltaic power conversion efficiencies exceeding 20%.^3^ This outstanding progress in photoelectric conversion has prompted the application of perovskite thin films in semiconductor devices. Organometallic-halide perovskites have been demonstrated in light-emitting diodes,^4^ optically pumped lasers,^5–7^ water splitting systems,^8^ and photodetectors.^9–15^

Numerous developments in high-performance perovskite solar cells have utilized mesostructured n–i–p TiO_2_/perovskite/2,2′,7,7′-tetrakis-(*N*,*N*-di-*p*-methoxyphenyl-amine)-9,9′-spirobifluorene (spiro-OMeTAD) semiconductor junctions. However, in optical sensing applications,^9,10^ devices with planar structures are attracting attention because they offer high pixel uniformity. Recent developments in perovskite photodiodes for optical sensing applications have thus focused on planar p–i–n configurations with organic hole transport layers.^12,13^ To fabricate p–i–n perovskite devices, indium tin oxide/poly (3,4-ethylenedioxythiophene):poly(styrene sulfonic acid) (ITO/PEDOT:PSS) substrates and [6,6]-phenyl C_61_-butyric acid methyl ester (PC_60_BM) are generally used. The perovskite layer is deposited by spin-coating onto the PEDOT:PSS layer as a light absorber, followed by the deposition of a thin PC_60_BM electron acceptor layer. Then, an Al cathode is typically deposited. However, various obstacles have so far prevented the realization of a homogeneous perovskite layer with a uniform thickness in planar heterojunction cells produced by solution processing. In order to solve the problems associated with the high roughness of thick perovskite films, thick (>110 nm) PC_60_BM layers can be used. Alternatively, another n-type metal oxide layer (such as TiO_*x*_) can be deposited onto a thin (∼50 nm) PC_60_BM layer to prevent shunting and reduce the leakage current under reverse bias by blocking direct contact between the perovskite film and the metal.^16,17^ To further improve the electronic properties of TiO_*x*_ materials processed at low temperatures (<150 °C), the introduction of extrinsic dopant elements is crucial. The modification of TiO_*x*_ has been successfully applied to dye-sensitized solar cells (DSSCs) and improved electron transport properties were observed in electron transport layers with optimal substitution of Y^3+^, Nb^5+^, and Ga^3+^.^18,19^ The doping of metal oxides has also been carried out to decrease interfacial recombination. In particular, the use of mixed Zr–TiO_*x*_ instead of pure TiO_*x*_ has been successful in improving the overall device performance of DSSCs and quantum dot solar cells.^20–22^ However, to the best of our knowledge, the effects of a Zr-doped TiO_*x*_ cathode interlayer on the performance of photodetectors have not yet been investigated in detail.

Hence, in this study, we demonstrate the performance of a photodetector developed with a Zr-doped TiO_*x*_ cathode interlayer. The photodetector was based on a p–i–n perovskite architecture, into which a Zr-doped TiO_*x*_ layer capped by Al was inserted. In order to explore the response speeds of our devices, we investigated the rise and decay times of the photocurrent using an oscilloscope with a pulsed laser diode as the light source. In addition, the capacitance and resistance components were carefully measured by impedance spectroscopy under dark and light conditions. The performance of the perovskite photodetectors with Zr-doped TiO_*x*_ layers is discussed herein, with a particular focus on detectivity and leakage current.

## Methods

### Fabrication of perovskite devices

A perovskite precursor (CH_3_NH_3_PbI_3−*x*_Cl_*x*_) was prepared as follows. We dissolved 420 mg of CH_3_NH_3_I (98%; Sigma Aldrich) and 245 mg of PbCl_2_ (99.999%; Sigma Aldrich) in 1 mL dimethylformamide (DMF) for over 4 h (ref. 23) and stored the resultant solution under vacuum to avoid moisture absorption. The configuration of the perovskite device was ITO/PEDOT:PSS/CH_3_NH_3_PbI_3−*x*_Cl_*x*_/PC_60_BM/TiO_*x*_/Al. ITO-patterned glass substrates were cleaned using acetone, isopropyl alcohol (IPA), and deionized (DI) water for 15 min each and then exposed to UV light and O_3_ for another 15 min. PEDOT:PSS (Heraeus Clevios AI4083) was spin-coated onto the ITO substrates at 5000 rpm for 60 s and annealed for 10 min at 150 °C on a hot plate. The perovskite precursor was deposited at 2000 rpm for 30 s and annealed for 90 min at 95 °C. A PC_60_BM layer (2% chlorobenzene solution; Nano Clean Tech Ltd.) was spin-coated at 1000 rpm for 30 s.

The TiO_*x*_ solution was prepared as follows. We mixed 369 μL of Ti-isopropoxide (99.999%, Sigma Aldrich) in 2.53 mL of IPA and 70 μL of 37% HCl in 2.53 mL of IPA in a 1 : 1 ratio.^24^ Zr-isopropoxide (99.9%, Sigma Aldrich) was added to the TiO_*x*_ solution in a weight ratio of 0–0.2%. The solution was stored in a refrigerator after each use. The TiO_*x*_ solution was spin-coated onto the PC_60_BM layer at 3000 rpm for 45 s and annealed at 130 °C for 10 min. A 100 nm-thick Al layer was deposited on the TiO*_x_* layer by a metal evaporator (VTR-300M/1ERH evaporator, ULVAC). The active surface area was 4 mm^2^. All fabrication steps, except for the deposition of the Al electrode, were carried out under glove-box conditions.

### Film characterization

The energy levels of the TiO_*x*_ (or Zr–TiO_*x*_) films were measured using an ultraviolet photo-electron spectrometer (UPS; Thermo Fisher Scientific Co., Ltd, USA) and an ultraviolet-visible (UV-vis) spectrometer (Jasco Co., Ltd., V-570). Surface morphology measurements were performed using a field-emission scanning electron microscope (FE-SEM; JSM-7100F, JEOL Co., Ltd.) and an atomic force microscope (AFM; NX 10, Park Systems Co., Ltd.). The Zr doping condition of the TiO_*x*_ film was measured by X-ray photoelectron spectroscopy (XPS; K-alpha, Thermo U.K. Co., Ltd.). Conductivity measurements were conducted using two-point measurements with two Al electrodes (∼30 nm thickness) on the TiO_*x*_ (or Zr–TiO_*x*_) films. The channel length was 200 μm and the channel width was 1 mm.

### Device characterization

Current–voltage curves and impedance spectra were measured using an Iviumstat (IviumStat Technologies, Netherlands) under AM 1.5G simulated solar radiation (Newport 69920 solar simulator, Newport Co., Ltd, USA) with a color filter (525 nm). The external quantum efficiency (EQE) was measured using a spectral incident photon-to-electron conversion efficiency (IPCE) system (Spectra Pro 300i, Acton research Co., Ltd) under monochromatic light generated by filtering the output from an O_3_-free Xe lamp and using a chopper frequency of 20 Hz. A calibrated Si photodetector was used to measure the incident monochromatic light intensity. The transient photocurrent (TPC) and dynamic characteristics were obtained using a photo-response measurement system (TNE Tech Co., Ltd). A light-emitting diode (525 nm) in the photo-response measurement system was modulated by a function generator to act as the excitation source. Square waves with different frequencies were applied. The photodetectors were directly connected to an oscilloscope (Tektronix TDS 3012B) with an input impedance of 50 Ω. The noise current was directly measured with a lock-in amplifier (Stanford Research Systems, SR830). The photodetectors were kept in a dark room and shielded by Al foils during measurements. The shot noise limit was calculated using 
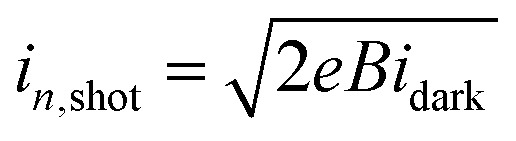
, where *e* is the elementary charge, *B* is the modulated bandwidth, and *i*_dark_ is the dark current.

## Results and discussion

### Organic–inorganic hybrid perovskite photodetector design

**Fig. 1:**
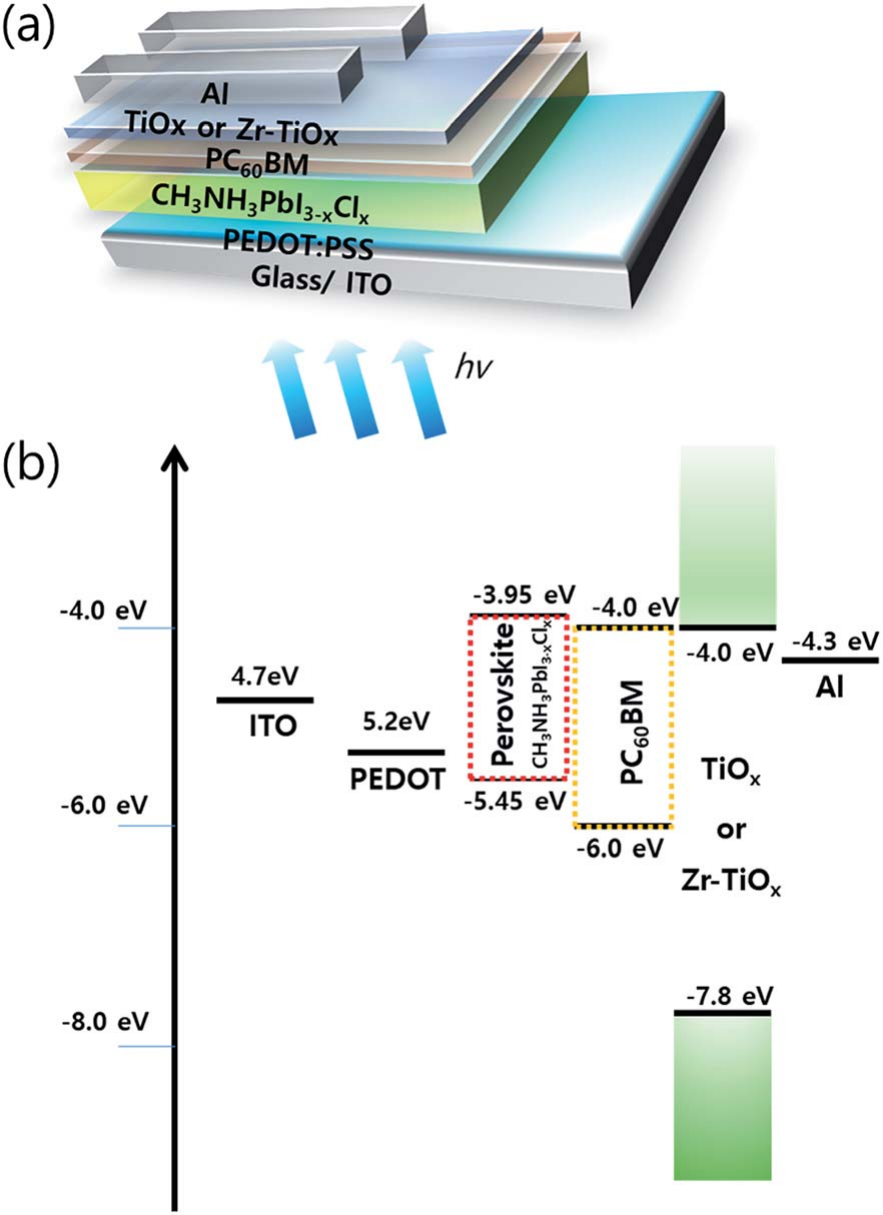
(a) Configuration of the perovskite photodetector with a TiO_*x*_ (or Zr–TiO_*x*_) cathode interlayer. (b) Energy diagram of the perovskite photodetector composed of ITO/PEDOT:PSS/CH_3_NH_3_PbI_3−*x*_Cl_*x*_/PC_60_BM/TiO_*x*_ or Zr–TiO_*x*_/Al.

The configuration and energy diagrams of our photodetector device are shown in Fig. 1(a) and (b), respectively. On top of the glass/ITO substrate, the organic–inorganic hybrid CH_3_NH_3_PbI_3−*x*_Cl_*x*_ layer is inserted between the PEDOT:PSS (p-type hole transport layer) and PC_60_BM (n-type electron transport layer). To reduce charge recombination and leakage current under reverse bias, TiO_*x*_ (or Zr–TiO_*x*_) was used as a hole-blocking material. Al was used as the top electrode. A cross-sectional SEM image of a typical perovskite photodetector employing a Zr–TiO_*x*_ layer is shown in Fig. S1† (which confirms the thickness of each layer of the photodetector). The energy levels of the TiO_*x*_ (or Zr–TiO_*x*_) film were confirmed using UPS (Fig. S2†) and UV-vis spectrophotometry (Fig. S3†). In the UPS data, the highest occupied molecular orbital (HOMO) and Fermi level of TiO_*x*_ are 4.08 eV and 7.8 eV and those of Zr–TiO_*x*_ are 4.0 eV and 7.8 eV, respectively. In the UV-vis data, the TiO_*x*_ and Zr–TiO_*x*_ exhibited similar bandgaps of approximately 3.8 eV. The Zr doping condition in the TiO_*x*_ film was confirmed through XPS (Fig. S4†). In the XPS map of the Zr–TiO_*x*_, a Zr 3d peak was identified at 182.5 eV and the intensity of the Ti 2p peak (at 458.5 eV) was decreased compared to the map obtained for the Zr-free TiO_*x*_. The O 1s core-level binding energy was observed at 530.0 eV.

### Detectivity characterization

The detectivity is related to the responsivity and noise-equivalent power (NEP; in W Hz^−1/2^). Responsivity (*R*(*λ*)) is given as the ratio between the generated photocurrent (*I*_ph_) and the amount of optical power (*P*_O_) incident on the detector, and is calculated from the EQE using the following equation:1
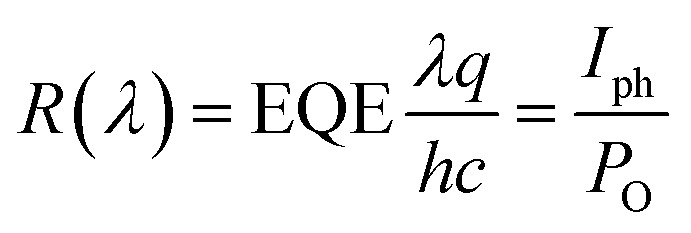
where *λ* is the wavelength, *q* is the electron charge, *h* is Planck's constant, and *c* is the velocity of light in vacuum. The NEP is defined as the incident optical power, where the signal and noise are quantified equally, and is calculated as follows.2
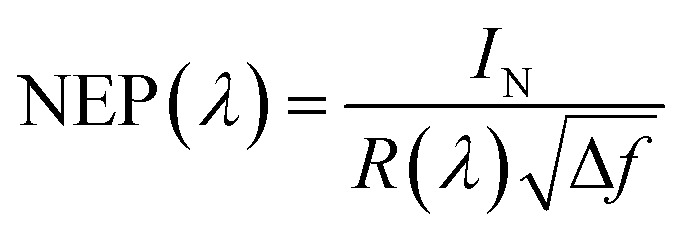
where Δ*f* is the bandwidth (Hz) and *I*_N_ is the total noise current. The reciprocal of the NEP is referred to as the detectivity of the device. The specific detectivity (D*) is given by the following equation:^25^3
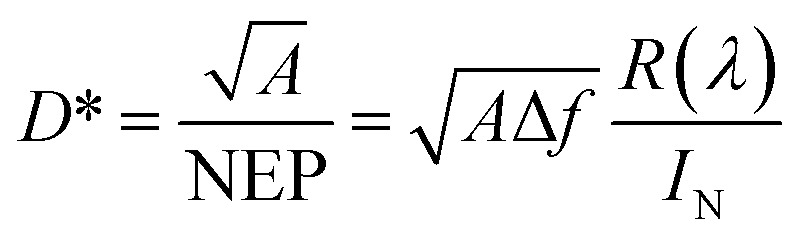
where *A* is the effective area of the detector and *f* is the electrical bandwidth. When the dark current is dominated by the shot noise, D* can be expressed as follows:4
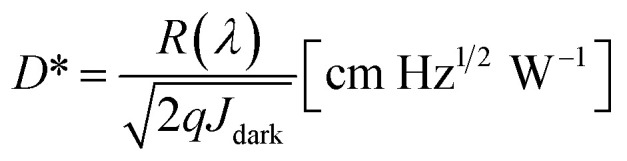
where *J*_dark_ is the dark current density, which should be kept as low as possible to obtain a high detectivity. In order to reduce the dark current, each thin film layer in the device should have a uniform surface and low trap concentration.

**Fig. 2:**
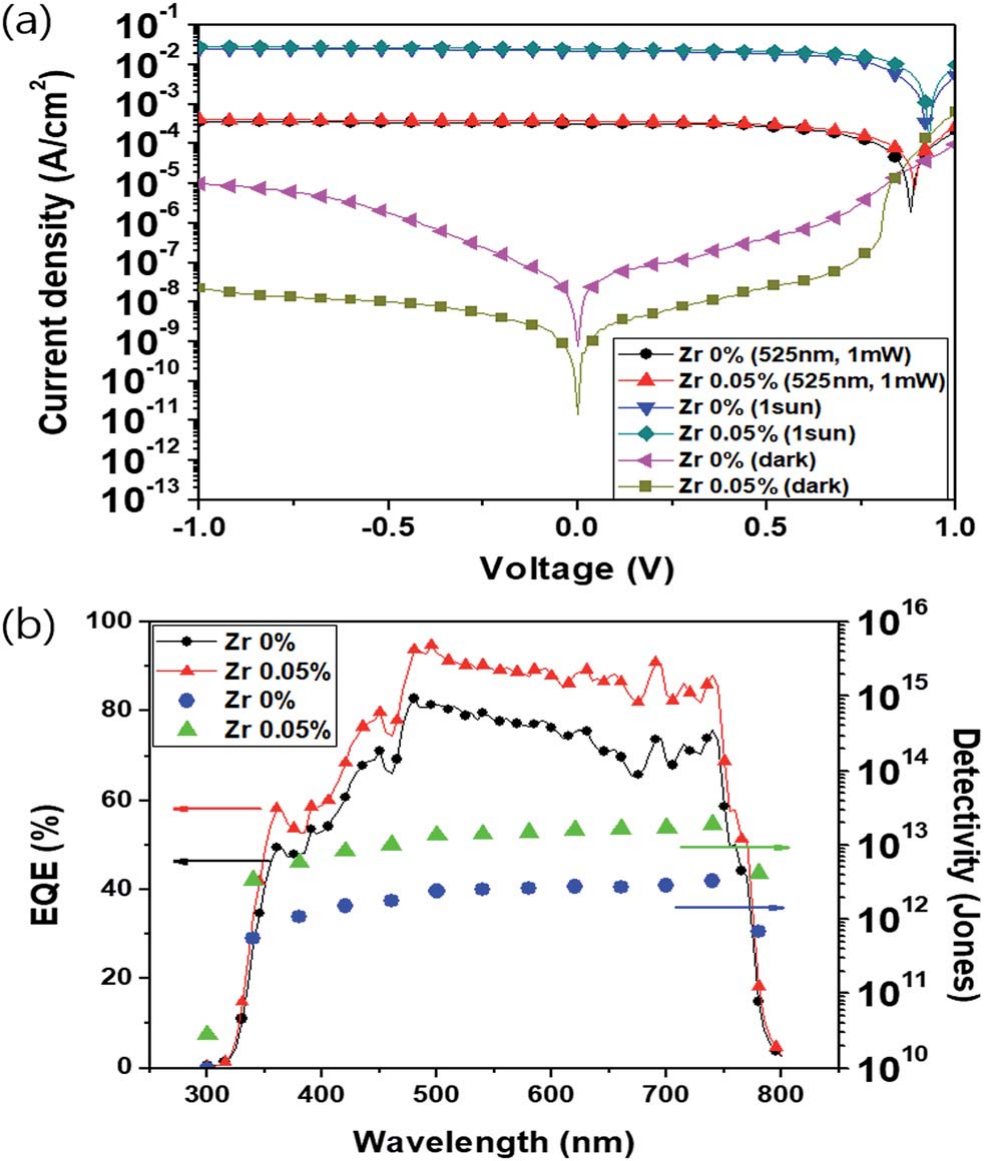
(a) Logarithmic *J*–*V* characteristics for perovskite photodetectors with (0.05%) Zr–TiO_*x*_ and Zr-free TiO_*x*_ cathode interlayers under 1 sun, dark, and 1 mW (525 nm) conditions. (b) External quantum efficiency (EQE) and detectivity of the perovskite photodetectors at different wavelengths (1 mW cm^−1^).

A comparison of the current density–voltage (*J*–*V*) parameters of TiO_*x*_ and (0.05 wt%) Zr–TiO_*x*_ is shown in Fig. 2(a). The *J*–*V* curve was measured in the dark and under illumination at *λ* = 525 nm (power density = 1 mW cm^−2^) and 1 sun. The photocurrent of the Zr–TiO_*x*_-based device increased slightly from 23.19 mA cm^−2^ to 25.59 mA cm^−2^ with 1 sun illumination and increased from 3.38 × 10^−4^ A cm^−2^ to 3.83 × 10^−4^ A cm^−2^ at 525 nm (1 mW cm^−2^) illumination as compared to the TiO_*x*_ reference device. Accordingly, the calculated responsivity of the Zr–TiO_*x*_-based device increased from 0.33 A W^−1^ to 0.38 A W^−1^ (Fig. S5†). The photocurrents remain almost constant at different reverse biases (0 to −1 V) and a very small voltage (−0.1 or even 0 V) could be applied to extract the electrons and holes generated in the detector. However, the dark *J*–*V* characteristics of the two different devices vary markedly. For the Zr-free device, the dark-current density of 5.92 × 10^−8^ A cm^−2^ (at −0.1 V) was measured. The specific detectivity at 525 nm was then calculated as 2.46 × 10^12^ Jones (where 1 Jones = 1 cm Hz^1/2^ W^−1^). Interestingly, the Zr-doped device shows a significantly reduced dark current of 2.43 × 10^−9^ A cm^−2^ (at −0.1 V) and a high specific detectivity of 1.37 × 10^13^ Jones as compared to the reference device. The hybrid perovskite photodetectors can also be self-powered and work at zero bias. The specific detectivity of the Zr-doped device at 0 V reaches 1.75 × 10^14^ Jones.

The concentration of the incorporated Zr was also studied to assess its influence on device performance (Fig. S6†). To achieve a statistically relevant result, we tested 30–32 nominally identical devices for each condition. The measured dark current (at −0.1 V) decreased significantly with the addition of 0.05 wt% Zr compared to the reference material and then showed similar values for the range between 0.05 wt% and 0.2 wt% Zr. The measured photocurrent density (at 1 sun and 525 nm, −0.1 V) and calculated specific detectivity (at 525 nm, −0.1 V) were maximized with the 0.05 wt% Zr composition with significantly lower values obtained upon increasing the weight ratio to 0.2%. This indicates that an excessively high concentration of Zr^4+^ is detrimental to the device photocurrent, possibly because it decreases the conductivity (Fig. S6(e)†). The EQE and detectivity of the Zr-doped and Zr-free devices at different wavelengths are shown in Fig. 2(b). The devices show photoresponses from 300 to 800 nm; the broad spectral responsivity of these photodetectors (Fig. S5†) indicates suitability for multispectral applications.^26^ The Zr-free device exhibits an EQE of 79%, while the Zr-doped device exhibits the highest EQE of 90% at 525 nm. From 350 to 750 nm, the detectivity of the Zr-doped device is approximately 1 × 10^13^ Jones, which is similar to that of a Si photodetector in the same spectral region.^27^

### Surface morphology

**Fig. 3:**
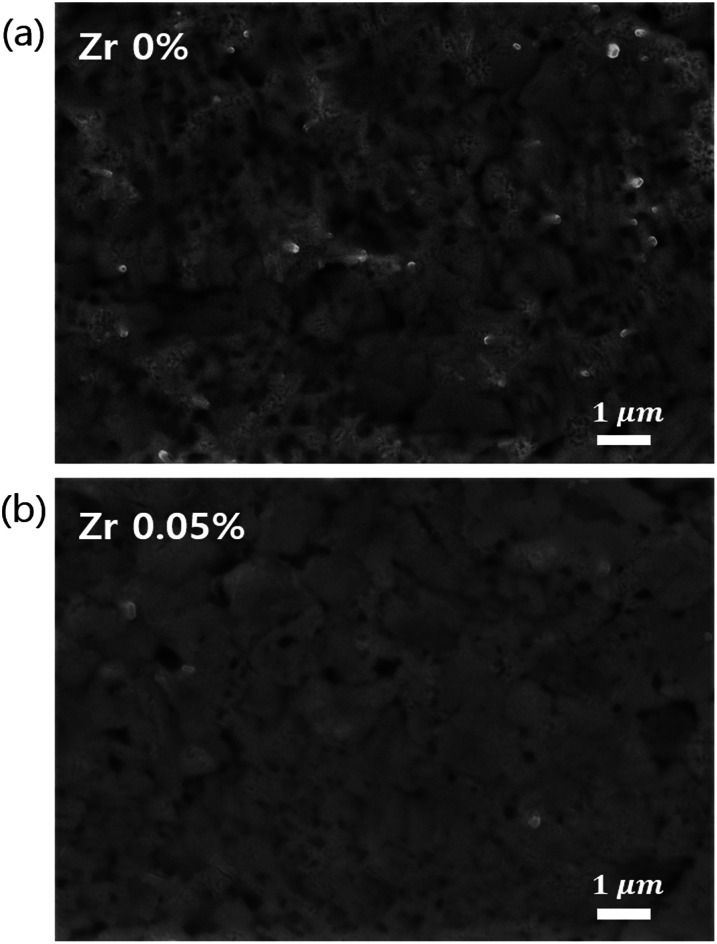
Top-view SEM images of (a) Zr-free TiO_*x*_ layer and (b) (0.05%) Zr–TiO_*x*_ layer deposited on perovskite/PC_60_BM layers.

The high detectivity of perovskite photodetectors primarily arises from their extremely low dark currents under reverse bias. Fig. 3 shows SEM images of reference TiO_*x*_ (Fig. 3(a)) and (0.05%) Zr–TiO_*x*_ (Fig. 3(b)) layers on the perovskite/PC_60_BM layer. The surface of the (0.05%) Zr–TiO_*x*_ layer (Fig. 3(b)) shows no defects and good coverage of the substrate, while the surface of the reference TiO_*x*_ layer shows numerous pinholes and cracks. In addition, the (0.05%) Zr–TiO_*x*_ film deposited on a Si wafer substrate showed no aggregates in AFM analysis unlike the reference TiO_*x*_ film (Fig. S7†). Pinholes and cracks in the TiO_*x*_ layer decrease its hole-blocking ability under reverse bias, thus increasing the dark current.

According to previous studies^28,29^ on metal-doped TiO_2_, Zr-doped TiO_2_ layer showed decreased nanocrystal sizes and suppressed nanocrystal aggregation because Zr-doping inhibited the densification and crystalline growth of TiO_2_ nanoparticles by providing dissimilar boundaries. For the same reason, TiO_*x*_ aggregation was suppressed by Zr doping in this study, contributing to the reduction of cracks and pinholes in the TiO_*x*_ layer. Hence, the Zr-doped device had a lower dark current than the Zr-free device.

### Dynamic characteristics

**Fig. 4:**
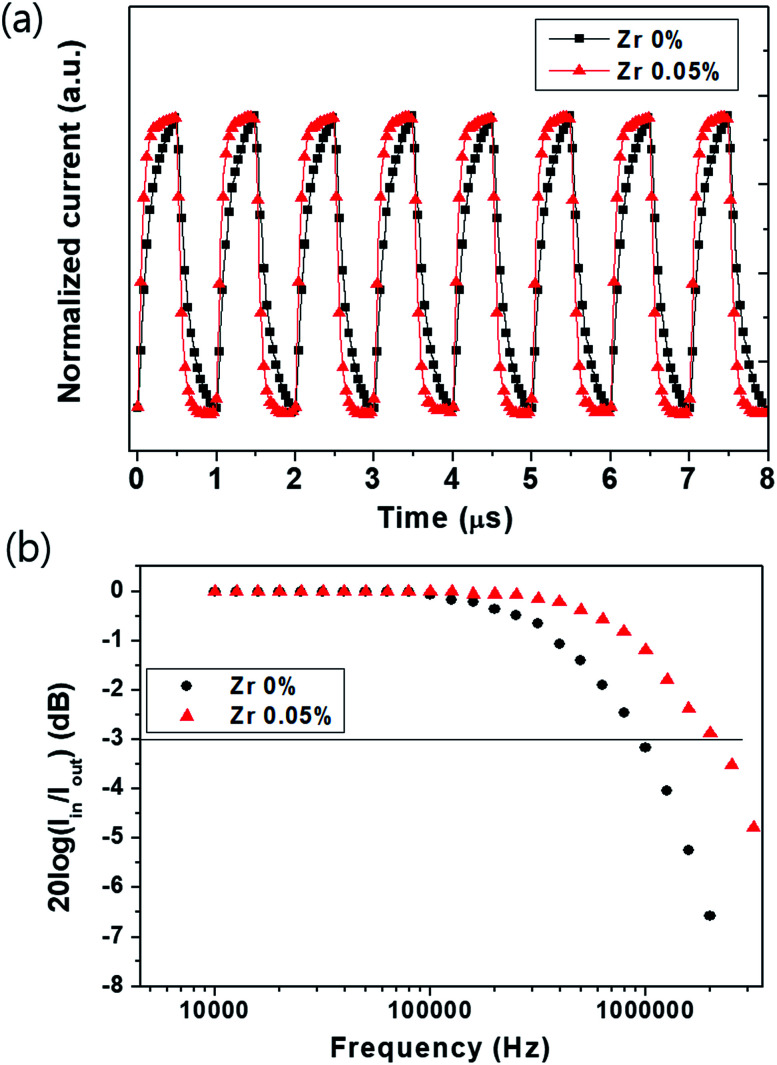
Dynamic characteristics of photocurrent response times using a laser diode at a light intensity of 650 μW cm^−2^ at 525 nm. (a) Photocurrent response time under −0.1 V at a pulsed frequency of 1 MHz. (b) Cut-off frequency for the perovskite photodetector under −0.1 V.

The response speed of a photodetector is closely related to charge transport and collection.^25^ Some devices must collect optical signals over a certain bandwidth (for example, high-frequency optical communication); this requires rapid extraction of photogenerated charge carriers. In order to explore the response speeds of our devices, we investigated the rise and decay times of the photocurrent using an oscilloscope and a pulsed laser diode as the light source. Fig. 4(a) shows the photocurrent response time of a few microseconds; this is defined as the characteristic time constant. The rise time (Δ*t*_r_) and decay time (Δ*t*_d_) can be expressed in terms of resistance characteristics^30^ as follows:

5 Δ*t*_r or d_ = *τ* ln 9 = *RC* ln 9

where *τ* is the characteristic time constant of the device, *R* is the resistance component, and *C* is the capacitance of the device. We measured the time taken to reach the highest point (90%) from the lowest point (10%) of the photocurrent and calculated the corresponding rise and decay times. The rise and decay times of the Zr-doped device are both 0.12 μs, which are shorter than those of the Zr-free device (Δ*t*_r_: 0.29 μs, Δ*t*_d_: 0.27 μs) at −0.1 V. The shape of the photocurrent response time is dependent on the resistance and capacitance behavior. The Zr-doped device shows lower series resistance and capacitance values than those of the Zr-free device. In particular, the (0.05%) Zr-doped TiO_*x*_ layer shows better surface uniformity on PC_60_BM than the undoped material, which promotes a higher shunt resistance.^31^ The resistance and capacitance of the photodetectors were analyzed *via* impedance measurements as discussed later.

We investigated the cut-off frequency to evaluate the figures of merit of the dynamic characteristics. The cut-off frequencies of the Zr-doped and Zr-free devices are shown in Fig. 4(b). In general, the bandwidth required for commercial applications is 0.1 MHz and the minimum required bandwidth for optical communication applications is 1 MHz.^32–34^ The bandwidth is calculated using the time constants, which are related to the response time. The bandwidth (with a cut-off frequency, *f*_c_, of −3 dB) is calculated using the following formula:

6 *f*_c_ = 1/(2π*τ*)

The bandwidth at −0.1 V of the Zr-doped device shows a frequency response of 2.1 MHz, which is higher than that of the Zr-free device (0.95 MHz). This result implies that photogenerated carriers in the perovskite layer of the Zr-doped device effectively reach each electrode without accumulation or recombination, thus increasing the photoresponse speed and bandwidth as compared to those of the Zr-free device.

### Trap density

In order to explore the physical effects of incorporating Zr into TiO_*x*_ on the performance of the perovskite-based photodetector, we investigated the densities of states (DoS) of the devices by measuring the transient photocurrent (TPC). We applied the method reported by Street^35^ to estimate the DoS from experimental TPC data.

**Fig. 5:**
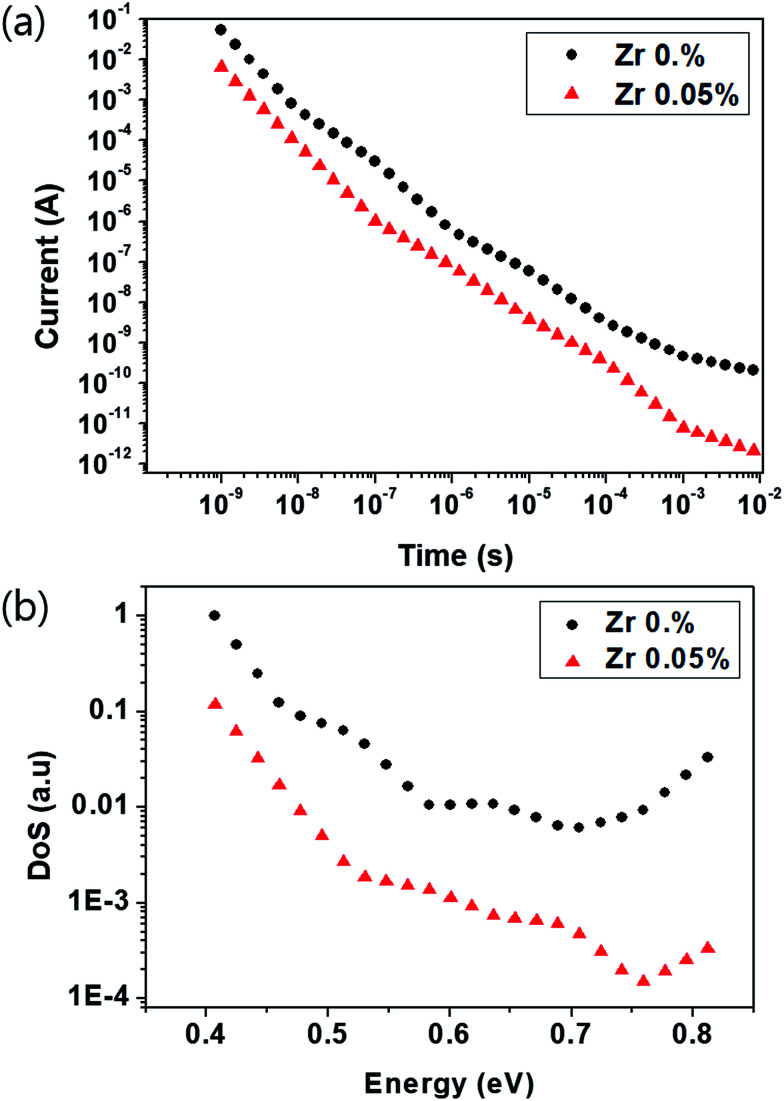
(a) Transient photocurrents of the device with the (0.05%) Zr–TiO_*x*_ layer and the reference device. (b) The DoS values extracted from the TPC values shown in (a).

The TPCs of the device with the (0.05%) Zr–TiO_*x*_ layer and the reference device are shown in Fig. 5(a) and the DoS results are shown in Fig. 5(b). As shown in Fig. 5(a), the Zr-doped device shows rapid photocurrent decay compared to the Zr-free device. According to the plots shown in Fig. 5(b), the trap density of the Zr-doped device is ten times lower than that of the reference device between 0.4 eV and 0.8 eV (where 0 eV is the TiO_*x*_ conduction level), indicating that the shallow traps of the TiO_*x*_ layer are passivated by the addition of Zr.

### Impedance analysis

**Fig. 6:**
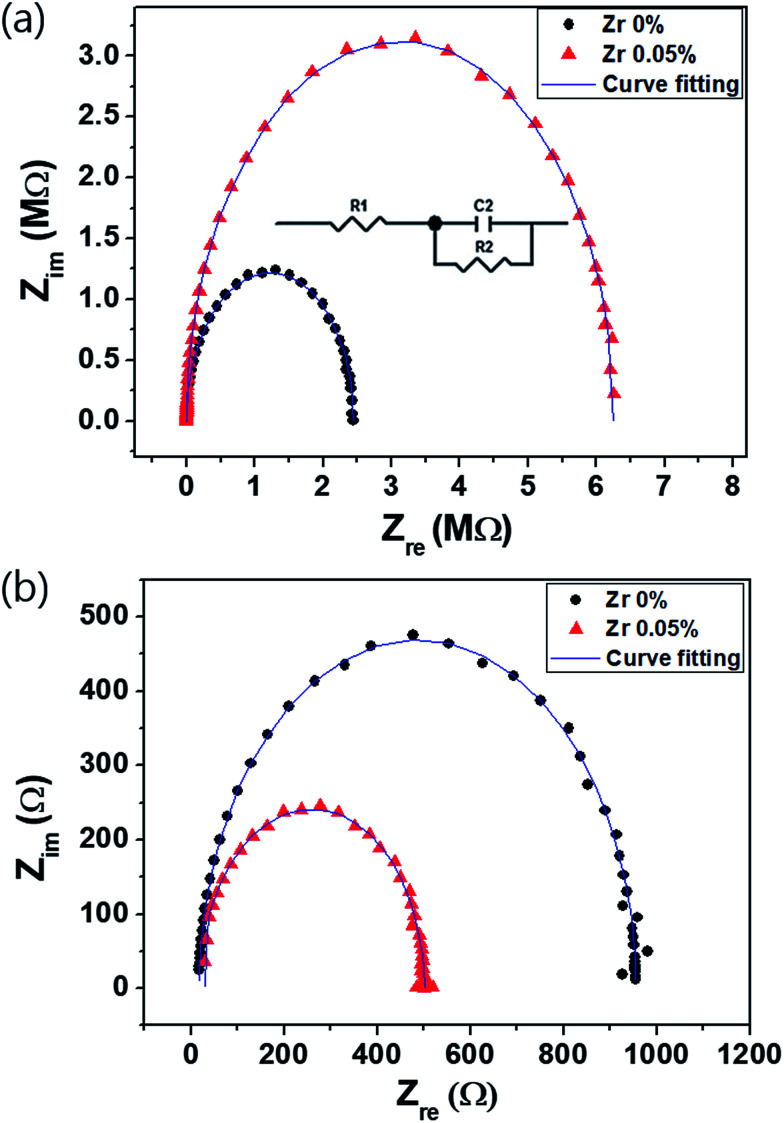
Impedance spectra measured (a) in the dark and (b) under illuminated conditions using a monochromator at 525 nm and 1 mW cm^−2^ at −0.1 V. The inset in (a) shows the equivalent circuit used to model the impedance.

To analyze the capacitance and resistance components of the multilayered devices, the impedance characteristics were measured by impedance spectroscopy under dark and illuminated conditions (at −0.1 V and 100 Hz to 30 MHz) (Fig. 6). Considering the device structure, the equivalent circuit shown in the inset in Fig. 6(a) is used as a model.^36^Table 1 lists the resistance and capacitance parameters from the fitting of each element in the equivalent circuit model, where *R*_1_ is the sheet resistance (20 Ω) through the ITO electrode and *R*_2_ and *C*_1_ are the resistance and capacitance of the device excluding the ITO electrode, respectively. In Fig. 6(a), *R*_2_ under the dark condition indicates the parallel resistance of the device, which is related to its leakage current. *R*_2_ under dark conditions is 6.25 × 10^6^ Ω for the device with the (0.05%) Zr–TiO_*x*_ layer, which is greater than that of the reference device (2.44 × 10^6^ Ω). The decrease in the leakage current of the Zr-doped device is attributed to its high parallel resistance. In addition, the dark current is primarily decreased by the blocking effect of the (0.05%) Zr–TiO_*x*_ as the cathode interlayer, which effectively prevents carrier injection, as shown by the *J*–*V* characteristics under the dark condition (Fig. 2(a)).

**Table 1:** Electrical parameters calculated from impedance values of perovskite photodetectors

	Dark		Light	
Zr content (wt%)	0	0.05	0	0.05
*R*_1_ (Ω)	20	20	20	20
*C*_1_ (F)	5.73 × 10^−10^	4.95 × 10^−10^	6.18 × 10^−10^	4.98 × 10^−10^
*R*_2_ (Ω)	2.44 × 10^6^	6.25 × 10^6^	940	486

The perovskite photodetector is also affected by the series resistance under illuminated conditions. The Zr-doped device shows the low *R*_2_ value of 486 Ω as compared to that of the Zr-free device (940 Ω) as shown in Fig. 6(b). Under illuminated conditions, *R*_2_ is related to the series resistance and depends on the active layer/electrode interfacial resistance. Hence, we confirm that the addition of Zr to the TiO_*x*_ layer reduces the contact resistance between the active layer and the electrode because of the uniform surface morphology of the (0.05%) Zr–TiO_*x*_ layer on the organic surface as shown in Fig. 3.

The capacitance is an important property determining the bandwidth and response of perovskite photodetectors. In terms of the *RC* circuit, capacitance affects the time constant (*τ*) and the rise time (Δ*t*_r_). Eqn (5) implies that a low device capacitance corresponds to a wide bandwidth and fast response. As shown in Table 1, the capacitance of the Zr-doped device is lower than that of the Zr-free device, which implies that the photo-generated carriers reach each electrode without accumulation or recombination. Therefore, the difference in capacitance is primarily attributed to the formation of passivated traps on the TiO_*x*_ layer surface as a result of the incorporated Zr.

### Linear dynamic range

**Fig. 7:**
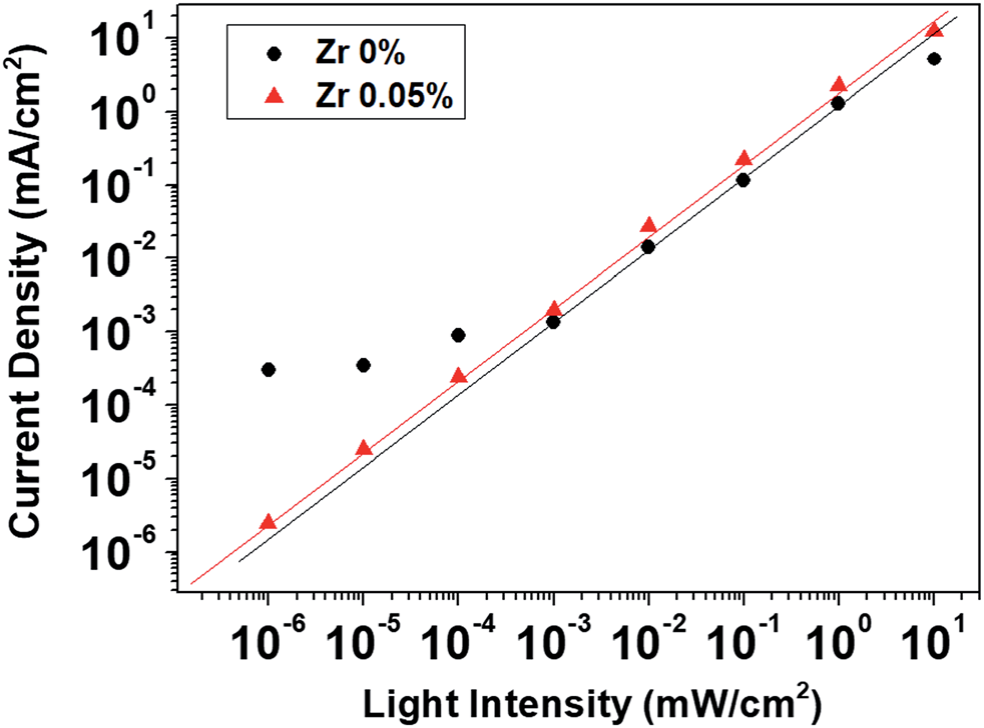
Current density as a function of light intensity (LDR) of perovskite photodetectors at −0.1 V.

We investigated the linear dynamic range (LDR or photo-sensitivity linearity, typically quoted in dB) in order to observe the response properties. The LDR is expressed as follows:7
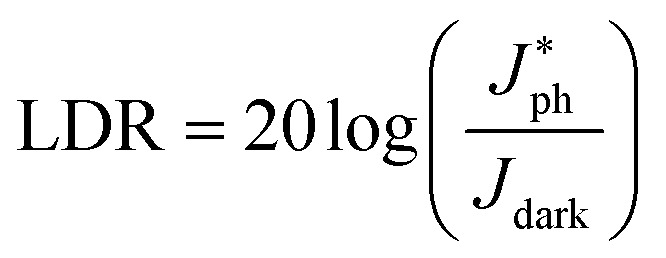
where 
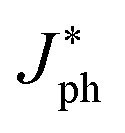
 is the photocurrent measured at 1 mW cm^−2^. Fig. 7 shows the photocurrent *versus* light intensity for devices with the (0.05%) Zr–TiO_*x*_ or reference TiO_*x*_ layers. When illuminated, the Zr-doped device shows a linear response over the incident light intensity range from 10^−6^ to 10^1^ mW cm^−2^ and an LDR of 119 dB. This range is comparable to that obtained for Si photodetectors (120 dB) and higher than those obtained for other types of photodetectors, such as InGaAs (66 dB).^13^ Thus, we confirmed that the hybrid perovskite photodetector with a (0.05%) Zr–TiO_*x*_ layer is suitable for the detection of a wide range of incident powers. Moreover, the reference device shows a linear response over a narrower incident light intensity range from 10^−3^ to 1 mW cm^−2^ and a low LDR of 86 dB, implying that the surface defects of the Zr-free TiO_*x*_ layer induce a large leakage current (or dark current) and low photocurrent in the device.

### Noise current and noise-equivalent power

**Fig. 8:**
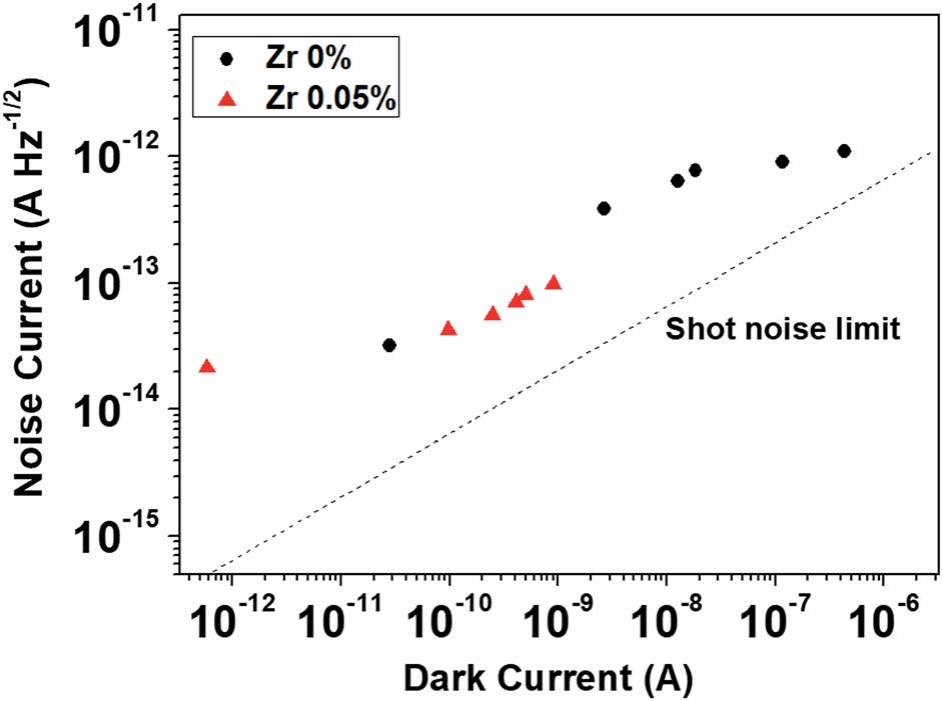
Noise currents of perovskite photodetectors at different dark currents. The shot noise limit is also plotted for comparison.

The NEP is another important performance index for optical detectors, representing the minimum incident optical power that a detector can identify as a photocurrent rather than noise. The NEP is defined as the optical signal power yielding a signal-to-noise ratio of 1 in a 1 Hz output bandwidth. It is equal to the noise current (*I*_N_; A Hz^−1/2^) divided by the responsivity (*R*; A W^−1^) (eqn (2)). To calculate the NEP values for the perovskite photodetectors, the noise currents of the devices were measured using a lock-in amplifier at a frequency of 100 Hz. Fig. 8 shows the noise currents obtained at different dark current levels; the noise current increases with an increase in dark current. The Zr-free device shows a larger noise current than that of the Zr-doped device, where the latter shows a small noise current of <0.1 pA Hz^−1/2^, which is about one order of magnitude smaller than that of a Si diode.^37^ The NEP of the photodetector was 1.12 × 10^−13^ W Hz^−1/2^ at 525 nm (−100 mV). Very small noise currents at low bias are critical for achieving such small NEP values for the perovskite photodetectors.

### Stability under ambient conditions

**Fig. 9:**
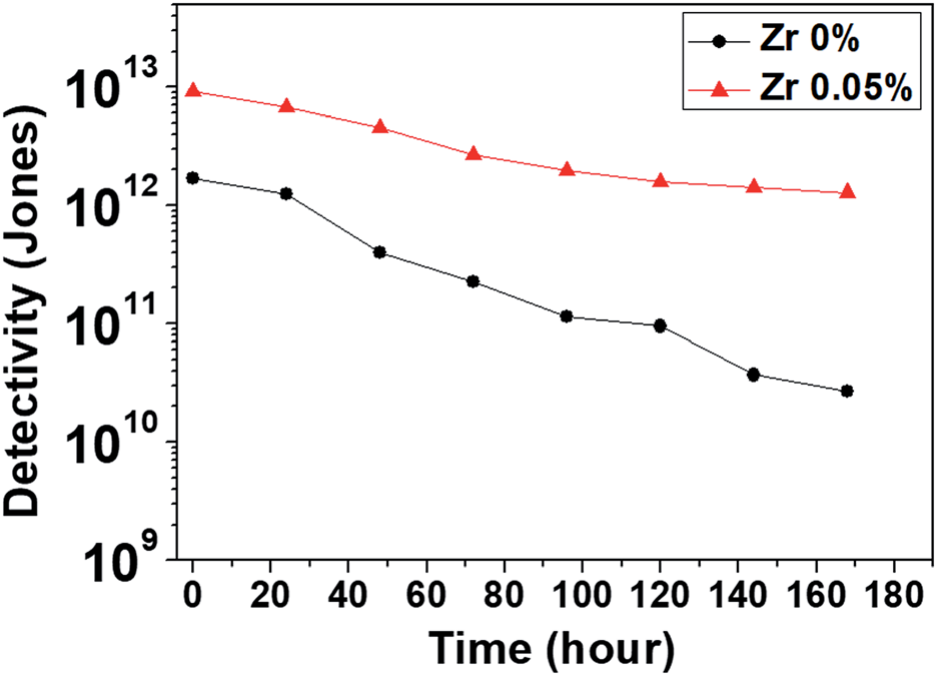
Detectivity of the perovskite photodetectors over 168 h (7 days) after fabrication under 1 sun of illumination (at −0.1 V).

The stability of the Zr-doped device was investigated in air over 168 h (7 days) after fabrication. In order to observe rapid changes, the devices were fabricated without encapsulation. Over 168 h, the devices were stored in the laboratory at ambient temperature. As shown in Fig. 9, the Zr-doped device shows more stable detectivity in ambient conditions than the reference device. The photocurrents and dark currents of the devices used for the calculation of D* values are shown in Fig. S8.† After 168 h, the Zr-doped device retains a D* of <10^12^ Jones (at 525 nm, −0.1 V), while that of the reference device decreased from 1.68 × 10^12^ to 2.69 × 10^10^ Jones under the same conditions.

The incompletely covered n-type layers on the perovskite film enable rapid chemical reactions by direct contact between the perovskite and Al electrode.^38^ Thus, the perovskite film was further exposed to the surrounding environment, which deteriorated the performance of the device. In addition, metals such as Al can diffuse more than 10 nm into the PC_60_BM layer during deposition.^39,40^ It can be concluded that the Zr–TiO_*x*_ layer effectively covered the PC_60_BM with neither defects nor pinholes, thus limiting the direct contact of the perovskite, PC_60_BM, and Al, O_2_, and moisture. Thus, the Zr-doped device exhibits high durability as compared to the Zr-free device.

## Conclusions

We demonstrated the behavior of (0.05%) Zr–TiO_*x*_ as the cathode interlayer of a perovskite photodetector. The device fabricated with a (0.05%) Zr–TiO_*x*_ layer showed a dark current density more than one order of magnitude lower than that of the Zr-free device. In particular, the surface defects of the TiO_*x*_ layer were suppressed by incorporating Zr, which improved the hole-blocking ability of the layer. Furthermore, the EQE of the Zr-doped device was 90% at 525 nm and exceeded that of the Zr-free device throughout the entire visible wavelength range. This increased efficiency arose from the surface trap passivation of the TiO_*x*_ layer by the added Zr, which improved carrier extraction. Thus, the Zr-doped device showed a high detectivity of 1.37 × 10^13^ Jones, which was about one order of magnitude larger than that of the Zr-free device. The Zr-doped device showed an impressive cut-off frequency of 2.1 MHz at −0.1 V despite a relatively low light intensity. A bandwidth greater than 1 MHz was observed, which was comparable with that of inorganic photodetectors based on Si^41–43^ and HgTe quantum dots,^44^ implying that the perovskite photodetector could be effectively applied to devices such as image sensors and in optical communication applications.

## Conflicts of interest

There are no conflicts of interest to declare.

## Supplementary Material

RA-008-C8RA00730F-s001
