# Correction: High-detectivity perovskite-based photodetector using a Zr-doped TiO_*x*_ cathode interlayer

**DOI:** 10.1039/c9ra90090j

**Published:** 2019-12-06

**Authors:** Chan Hyuk Ji, Kee Tae Kim, Se Young Oh

**Affiliations:** Department of Chemical and Biomolecular Engineering, Sogang University Seoul Korea syoh@sogang.ac.kr +82-2-714-3890 +82-2-705-8681

## Abstract

Correction for ‘High-detectivity perovskite-based photodetector using a Zr-doped TiO_*x*_ cathode interlayer’ by C. H. Ji *et al.*, *RSC Adv.*, 2018, **8**, 8302–8309.

The authors regret that the names of the authors are shown incorrectly in the original article. The corrected author list is as shown above.

In addition, the authors regret that an incorrect version of Fig. 4b was included in the original article. The correct version of Fig. 4 is as shown below.

**Fig. 4:**
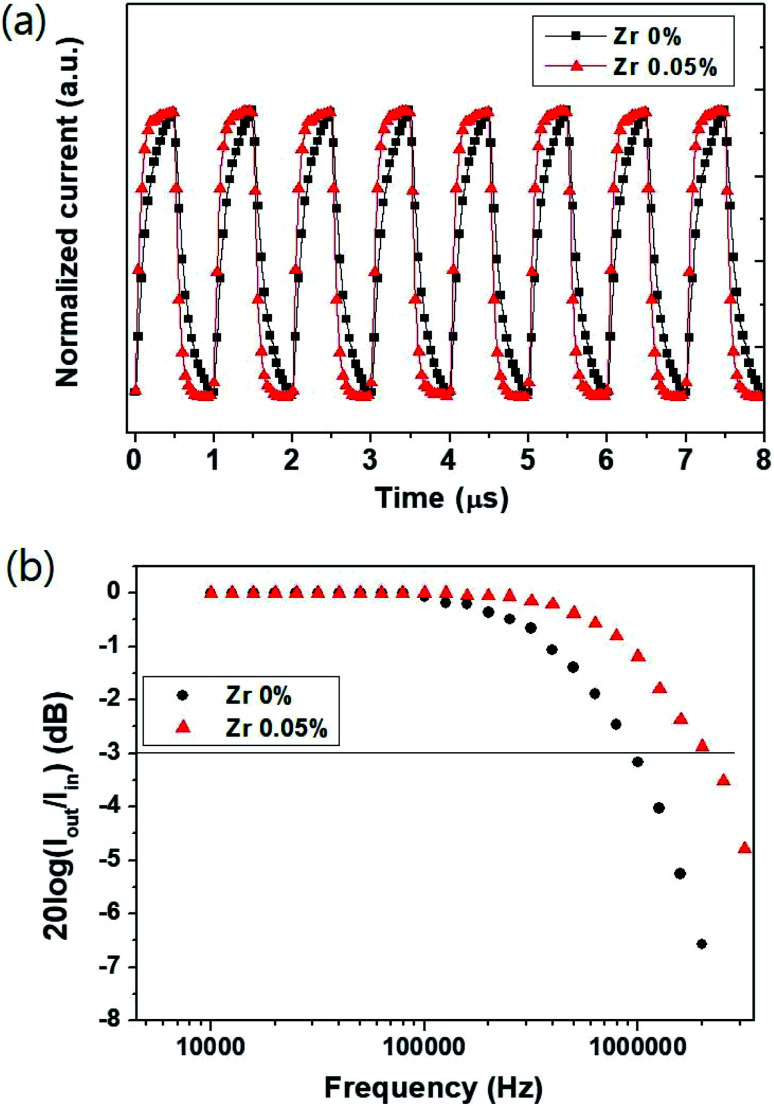
Dynamic characteristics of photocurrent response times using a laser diode at a light intensity of 650 μW cm^−2^ at 525 nm. (a) Photocurrent response time under −0.1 V at a pulsed frequency of 1 MHz. (b) Cut-off frequency for the perovskite photodetector under −0.1 V.

The Royal Society of Chemistry apologises for these errors and any consequent inconvenience to authors and readers.

## Supplementary Material

