# Supplementary material for: High-detectivity perovskite-based photodetector using a Zr-doped TiOx cathode interlayer
Source: RSC Adv. 2018 Feb 22;8(15):8302–9. doi: 10.1039/c8ra00730f (PMC9078570; doi:10.1039/c8ra00730f)
Supplement: RA-008-C8RA00730F-s001 [file RA-008-C8RA00730F-s001.pdf]

Electronic Supplementary Material (ESI) for RSC Advances.
This journal is © The Royal Society of Chemistry 2018

High-detectivity perovskite-based photodetector using a Zr-doped TiO_x cathode interlayer

C. H. Ji, K. T. Kim and S. Y. Oh

Supporting Information

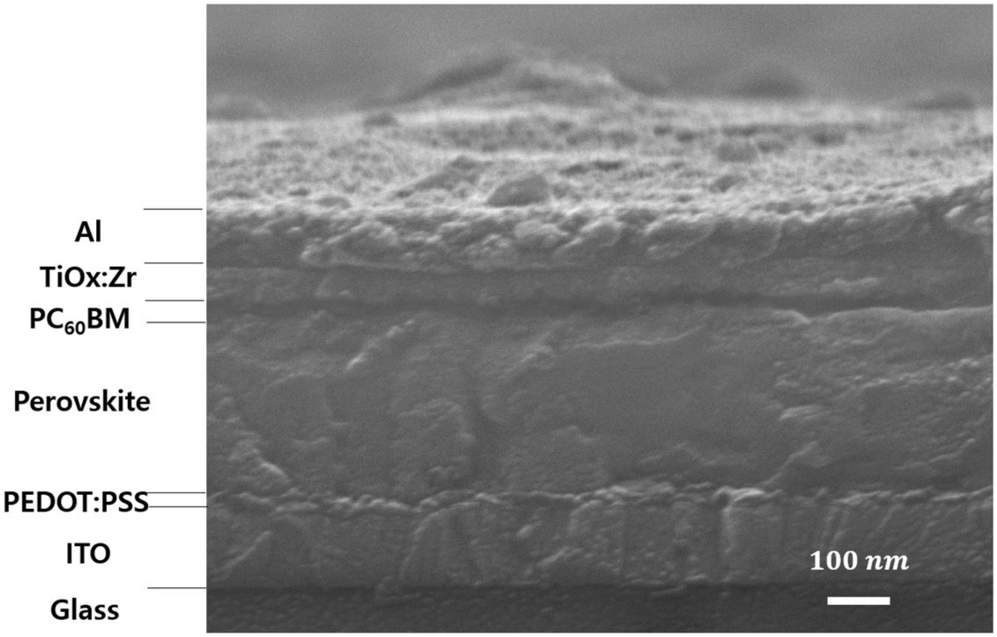

Figure S1. Cross-sectional scanning electron microscopy images of the perovskite photodetector with (0.05%) Zr-TiO_x layer.

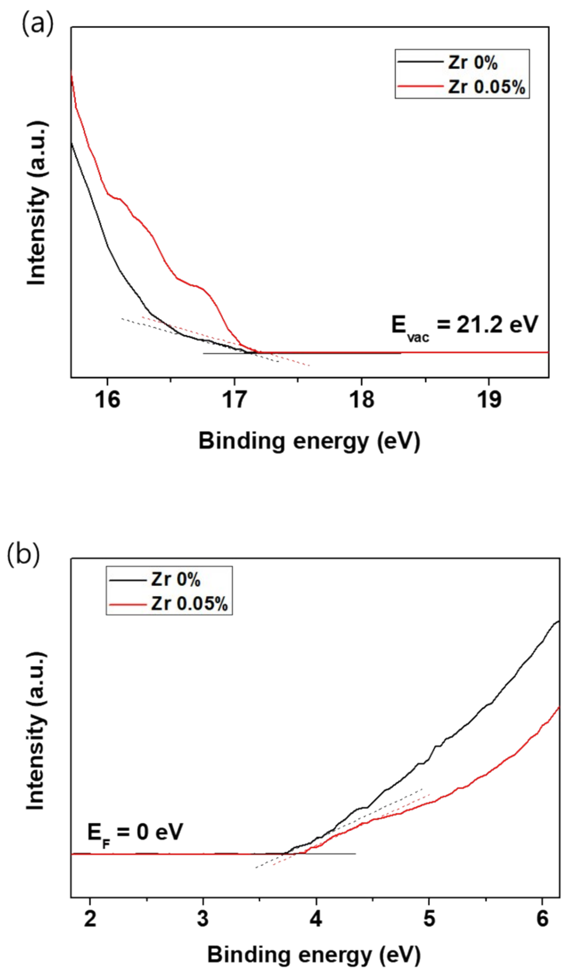

Figure S2. Photoemission cut-off obtained via UPS for TiO_x with and without Zr. (a) Cut-off region and (b) HOMO region. Fermi energy (E_F) levels for cathode interlayers: 4.08 eV for Zr-free TiO_x and 4.0 eV for (0.05%) Zr-TiO_x. HOMO levels for cathode interlayers: 7.8 eV for Zr-free TiO_x and 7.8 eV for (0.05%) Zr-TiO_x.

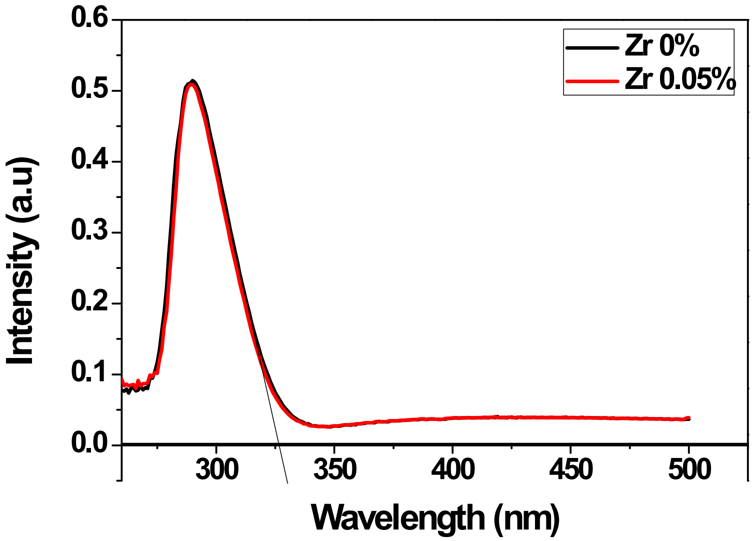

Figure S3. UV-visible absorption spectra of TiO_x with and without added Zr, where the extrapolated line is used to calculate the optical bandgap (all the cathode interlayer materials exhibit similar bandgaps of approximately 3.8 eV).

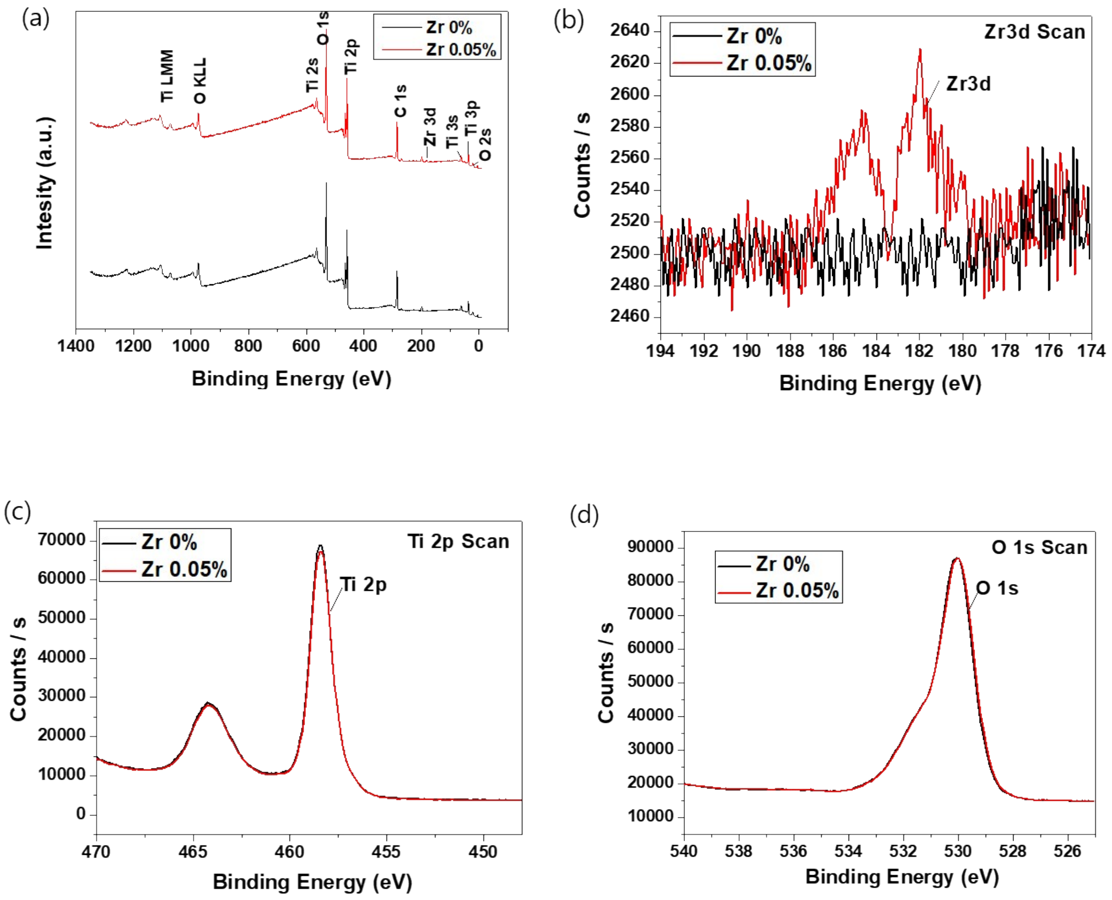

Figure S4. X-ray photoemission spectra for the Zr-free TiO_x layer and 0.05% Zr-TiO_x layer on glass substrates. (a) Wide-angle survey spectra, (b) Zr 3*d* orbital, (c) Ti 2*p* orbital, and (d) O 1*s* orbital. We confirmed the incorporation of Zr into the TiO_x layer by identifying the Zr 3*d* feature at 182.5 eV and the decrease in intensity of the Ti 2*p* feature at 458.5 eV. We also observed O 1*s* core-level binding energies at 530.0 eV.

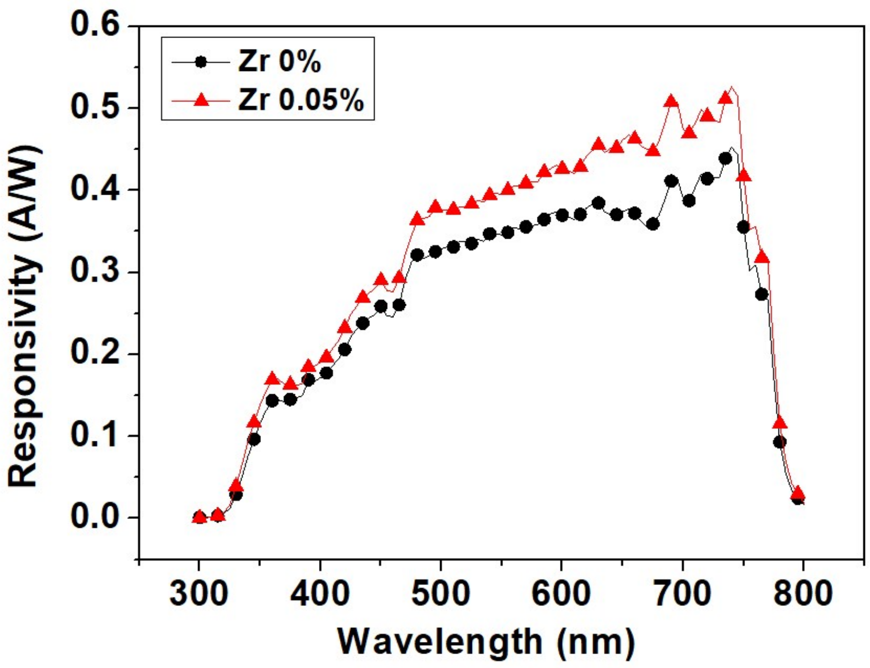

Figure S5. Responsivity of the perovskite photodetectors based on TiO_x cathode interlayers with and without added Zr at different wavelengths (1 mW/cm).

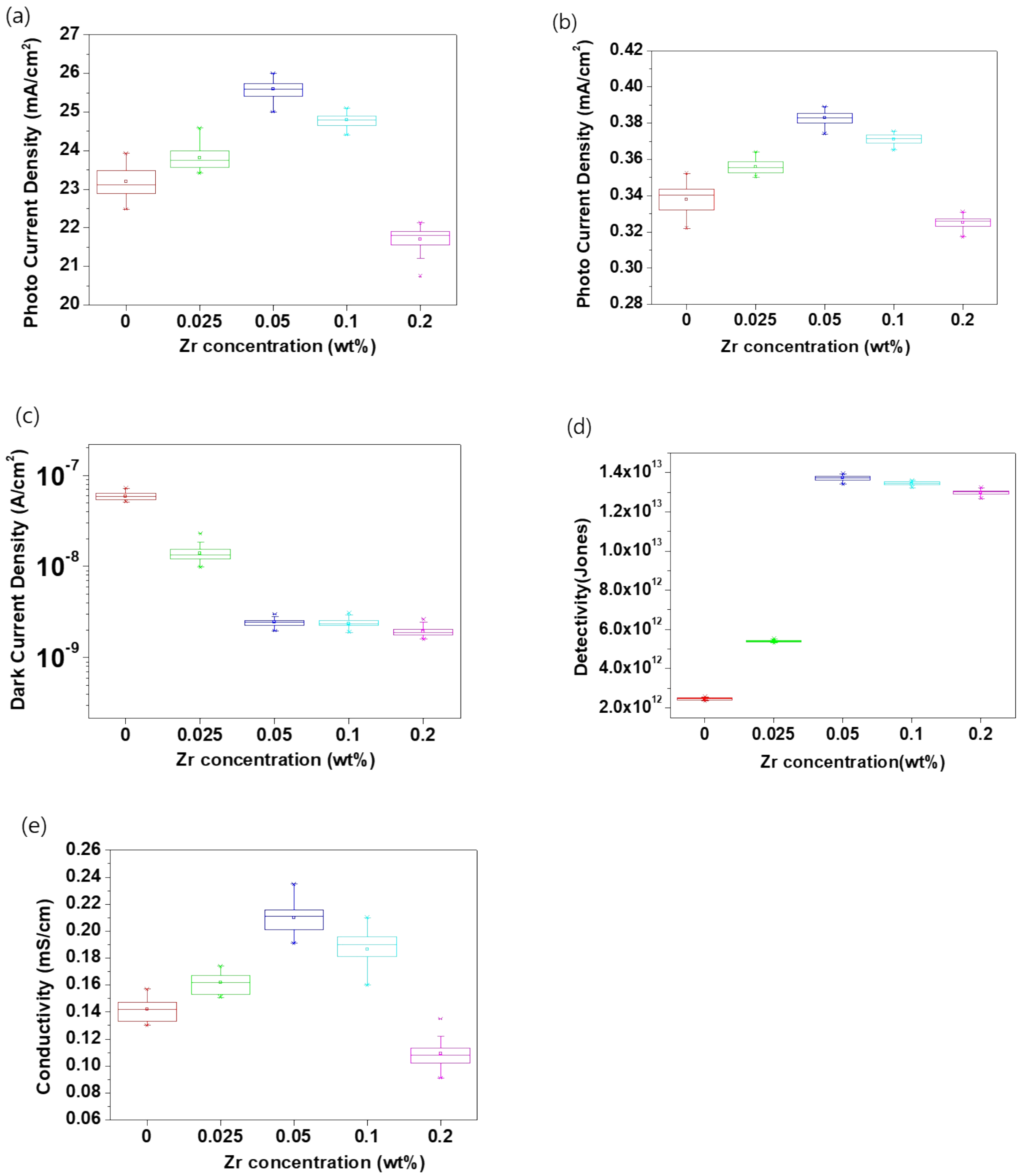

Figure S6. Device statistics for (a) photocurrent density (at 1 sun, −0.1 V), (b) photocurrent density (at 525 nm, −0.1 V), (c) dark-current density, (d) detectivity, and (e) conductivity of perovskite photodetectors employing the cathode interlayers with different Zr concentrations. The standard deviations of the data are represented by standard box plots (90% of all data points fell between the upper and lower whiskers).

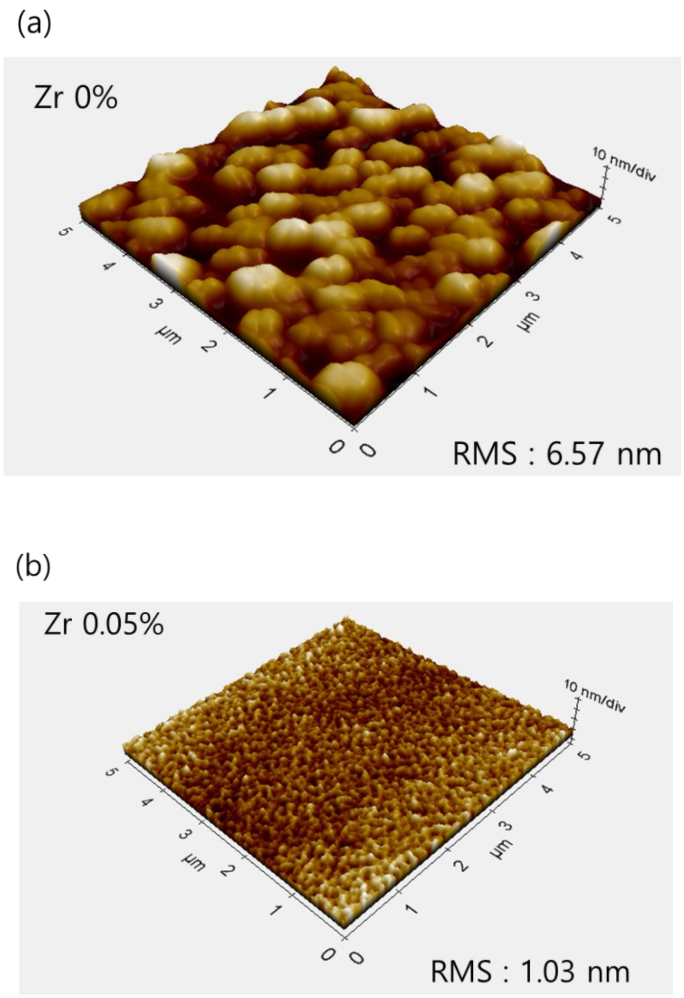

Figure S7. AFM images of the (a) Zr-free TiO_x and (b) (0.05%) Zr-TiO_x layers on Si wafer substrates.

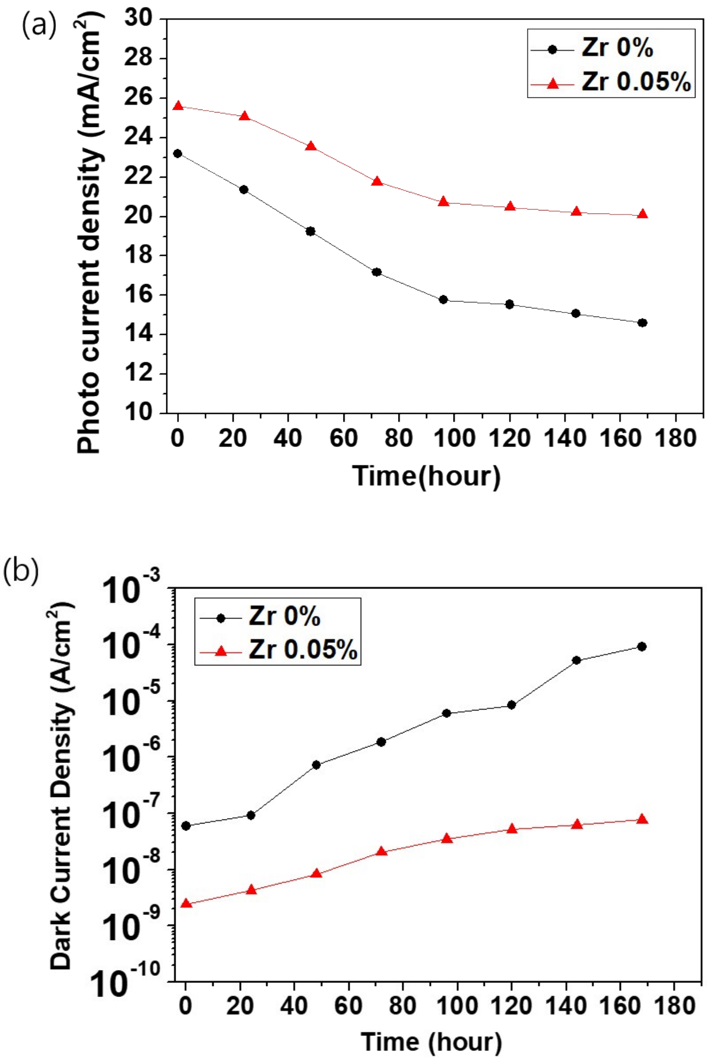

Figure S8. (a) Photocurrent densities (under 1 sun of illumination) and (b) dark-current densities of perovskite photodetectors over 168 h (7 days) after fabrication.
